# Rewiring Young Minds: Investigating the Cognitive Effects of Video Games on Learning and Their Potential as Digital Therapeutics for Mental Well-Being

**DOI:** 10.7759/cureus.87414

**Published:** 2025-07-07

**Authors:** Enoch Chi Ngai Lim, Nga Chong Lisa Cheng, Chi Eung Danforn Lim

**Affiliations:** 1 Research and Development, Specialist Medical Services Group, Earlwood, AUS; 2 National Institute of Complementary Medicine (NICM) Health Research Institute, Western Sydney University, Sydney, AUS; 3 School of Life Sciences, University of Technology Sydney, Sydney, AUS

**Keywords:** adhd, autism spectrum disorder, cognitive development, digital therapeutics, educational gaming, mental well-being, video games

## Abstract

Video games are commonly integrated into the lives of childhood and adolescence, with their impact on cognition and mental health widely debated. Concerns about academic distraction are increasingly balanced by recognition of their potential cognitive and therapeutic value. This review aims to explore the dual role of video games in pediatric populations, examining both their potential cognitive risks and their emerging utility as digital mental health interventions. It examines two key roles that video games may play in a child’s life: as potential risk factors for academic distractibility and as emerging digital interventions for mental health conditions. While video games may enhance attention, executive control, and working memory, excessive use can disrupt sleep patterns, reduce focus, and hinder academic performance. Therapeutic applications of video games are gaining interest, with certain games developed to enhance cognitive function in attention-related conditions and others aimed at fostering social and emotional learning in children with developmental differences. Immersive platforms are also being explored for emotional regulation and exposure-based therapies. Healthcare professionals should guide parents and educators to recognize both the potential benefits and risks of video game use. The future of therapeutic gaming lies in the integration of AI, personalized interventions, structured clinical applications, and interdisciplinary collaboration among neuroscientists, clinicians, educators, and game developers.

## Introduction and background

Video games are now widely played across all age groups, but they remain a particularly prominent part of childhood and adolescence. Many young individuals engage with video games on a daily or near-daily basis, making gaming a regular feature of their routines. The widespread popularity of video games has raised discussions and controversies regarding their impact on cognitive learning and mental health. In Australia, video gaming is deeply embedded in the lives of children and adolescents, making it a frequent subject of educational and psychological research. This widespread engagement highlights the importance of understanding both the developmental risks and potential cognitive or therapeutic benefits. Excessive video gaming has been associated with several adverse effects, including attention deficits and mood disorders. However, a study with a sample of almost 2000 children showed that those gamers who played video games for three or more hours per day scored higher on memory and impulse-control tasks than non-gamers [1]. This review explores how video gaming influences cognitive abilities related to learning and examines its growing role as a digital therapeutic tool for youth mental health. By reviewing both the risks and advantages of playing video games, healthcare professionals could support parents in achieving the ideal balance between gaming benefits and risks.

## Review

Methodology

This article employs a narrative review approach to synthesize the literature on the cognitive and therapeutic effects of video games on children and teenagers. Research articles, systematic reviews, randomized controlled trials, and meta-analyses published from 2005 to 2024 were obtained through PubMed, PsycINFO, Scopus, and Google Scholar. The search utilized the following keywords: “video games”, “cognitive development”, “ADHD”, “autism spectrum disorder”, “digital therapeutics”, “mental health”, and “children”. The inclusion criteria focused on the age range of five to 18 years, therapeutic or cognitive digital games with psychological or educational outcomes, and therapeutic gameplay with quantifiable results. Exclusion criteria were non-peer-reviewed literature, violence-centric entertainment gaming with no therapeutic value, and entertainment-focused games lacking cognitive elements. The literature was analyzed and organized into two main themes: (1) cognitive impacts of video games on learning and attention and (2) their use as therapeutic tools for various mental health disorders. This methodology adds structure while maintaining flexibility to capture the state of the art in this rapidly evolving area. While no formal risk of bias assessment was conducted, higher-level evidence (e.g., meta-analyses and RCTs) was prioritized where available. Themes were synthesized based on patterns of consistency and divergence across studies, with attention to reported outcomes and contextual factors relevant to the pediatric population.

Cognitive impact of video games on learning

The effects of video games on cognition are extensive, and some genres can serve as active brain training. For example, action games can enhance visual-spatial capacities, including spatial navigation, mental rotation, and attentional control. A meta-analysis of action video games revealed that the positive influence of video games extends to various cognitive functions, including working memory, selective attention, and processing speed [2]. Notably, regular gamers outperform non-gamers in tasks that require rapid decision-making and visuospatial reasoning. Short-term gaming interventions also support cognitive improvement: as little as 10 hours of playing an action game can improve spatial attention in naïve players [2]. Longitudinally, adolescents who often play strategy video games show improved problem-solving skills over time, which may enhance their academic performance [3]. Such findings align with the idea that strategy-based games require players to plan, manage resources, and adapt to new challenges, thereby reinforcing higher-order cognitive skills. Similarly, puzzle games have been associated with improvements in logical reasoning and pattern recognition, and simulation games may promote decision-making and planning by mimicking real-life systems in educational contexts.

Despite these possibilities, the risks associated with gaming have a negative impact on cognition, learning, attention, and distraction. Research indicates that longer gaming sessions are linked to reduced attention spans in children, including difficulties maintaining focus and increased impulsivity [4]. In a longitudinal study, bidirectional negative effects were also observed: more time spent gaming led to increased attention difficulties in children, while children who already experienced attention and impulse-control problems were more likely to engage in excessive gaming, thereby reinforcing their cognitive challenges [4]. This cyclical relationship suggests that children with underlying vulnerabilities may be both more attracted to and more affected by gaming, potentially exacerbating difficulties with sustained focus, self-regulation, and academic engagement over time. Time displacement, the concept of gaming hours reducing time spent on sleeping, reading, or doing homework, is another risk. One study supports this displacement, finding that adolescent gamers read 30% less and complete 34% less homework compared to non-gamers [5]. Another closely related issue is that long sessions or late-night gaming can disrupt adolescent sleep. Screen devices used in video games are strongly related to short sleep duration and bedtime delays in youth [6]. This is achieved through mechanisms such as sleep time’s space displacement, mental engagement from interactive play, and bright light exposure at night, which can suppress melatonin. Chronic sleep deprivation negatively influences learning, memory consolidation, and emotional regulation.

**Figure 1:**
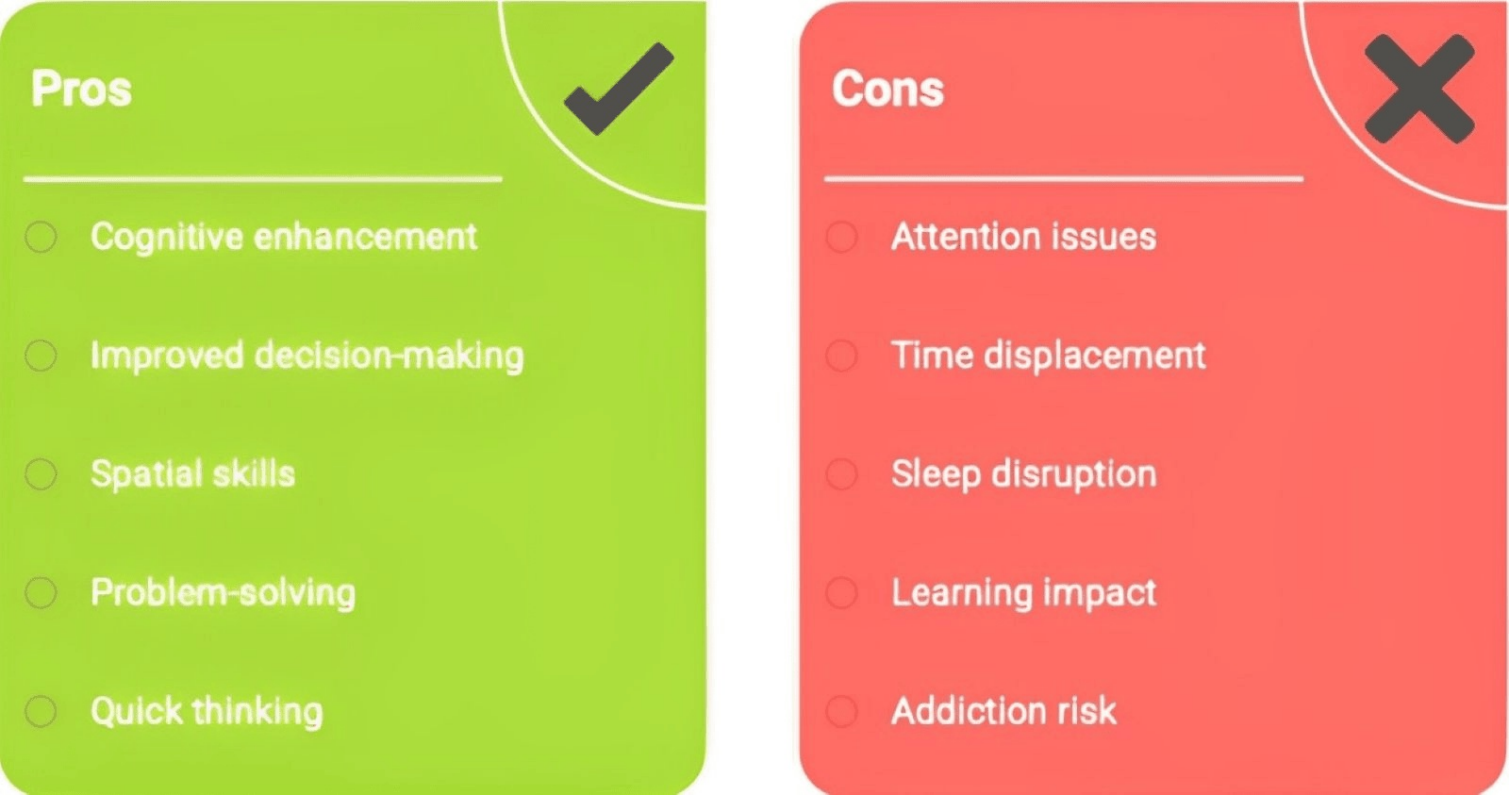
Pros and cons of playing video games

Video games have both compelling pros and cons for cognitive development, as illustrated in Figure 1. Action and strategy games may improve specific cognitive capacities associated with learning, such as spatial attention, quick thinking, and problem-solving. However, excessive or unstructured gameplay can be detrimental to attention span, potentially replacing homework time and healthy sleep, which are essential for optimal cognition and development. Overall, the final effect depends on how an individual child manages gaming. 

Video games as digital therapeutics for mental health

Video games are increasingly used for both entertainment and as therapeutic aids in the treatment of psychological disorders and as a form of mental health support. Digital therapeutics, also known as “serious games,” utilize their interactivity to offer therapeutic interventions in the form of enjoyable games. This subsection evaluates research supporting video game intervention proposals for multiple youth mental health disorders, as illustrated in Table 1.

**Table 1:** Video game interventions for youth mental health conditions ADHD, attention-deficit/hyperactivity disorder; ASD, autism spectrum disorder; CBT, cognitive behavioral therapy

Characteristic	ADHD	ASD	Other mental health conditions
Therapeutic goal	Improve focus and self-control	Promote social and emotional skills	Confront fears and learn to cope
Game examples	EndeavorRx (AKL-T01)	Secret Agent Society (SAS)	MindLight, SPARX, VR Simulations
Therapeutic approach	Adaptive algorithms and multitasking	Parental guidance and collaboration	CBT-based exposure therapy, biofeedback
Efficacy	Greater attention than placebo	Increased social skills	Reduced anxiety and depressive symptoms

Attention-Deficit/Hyperactivity Disorder (ADHD)

ADHD is a congenital neurodevelopmental disorder that is indicated by symptoms of inattention, hyperactivity, and impulsivity. One modern therapeutic approach is the development of video games that can be used as an adjunct or alternative therapy approach to help address the cognitive deficits associated with ADHD. In 2020, the FDA approved the first-ever prescription video game for ADHD in children. The video game “EndeavorRx,” previously known as “AKL-T01,” is an immersive racing video game that utilizes multitasking challenges and adaptive algorithms to enhance children’s attention. An RCT found that children with ADHD who played the therapeutic video game for 25 minutes a day, five days a week, over four weeks showed improved attention compared to those using a placebo version of the game [7]. The children who played the therapeutic video game exhibited improved performance on the Test of Variables of Attention (TOVA), an objective measure of attentional control; however, their TOVA scores failed to show significant increases. Game-based interventions help children improve their focus and self-control, and the improvement of attention is facilitated by the fact that the games reward players for maintaining focus and self-discipline. A successful result from EndeavorRx demonstrates how prescribed video games could become an element in ADHD treatment to provide an interactive, non-drug remedy for young patients.

Autism Spectrum Disorder (ASD)

Social skills and communication challenges, along with increased anxiety, are characteristic of many children with autism [8]. Video games enable children with ASD to learn social skills and acquire new competencies in a controlled environment where stressors from the real world are absent. The Secret Agent Society (SAS) is one of several game interventions designed to promote social and emotional skills in children with autism [8]. In a 10-week randomized study, the SAS game, which involved parental guidance, helped to increase the social skills of adolescents (aged seven to 12) with ASD compared to an active control condition group [9]. Teachers identified improved social skills as a positive factor in children who played SAS games, while positive social interactions and fewer problem behaviors were apparent from observations of parents [9]. Game experiences enable children with ASD to learn and practice emotion regulation and social problem-solving while collaborating with peers in an engaging environment.

Many children with ASD find themselves extremely anxious because of their sensory sensitivities and social circumstances, which cannot be controlled, leading to an increase in stress. Games that incorporate relaxation training and gradually expose children to their fears show promising therapeutic potential for children with autism. For example, calming environments for games have been developed by researchers (such as “New Horizon Game,” a serious game that features built-in relaxing games) that encourage players to be in charge of their feelings of stress. As a result of the pilot studies conducted on these games so far, the children have demonstrated a clear improvement in their stress and anxiety levels [10]. These types of therapeutic gaming procedures encourage children to practice breathing exercises or mindfulness during gameplay, allowing for the reduction of arousal and learning important aspects of coping in an enjoyable way. However, these are only small-scale, early advances in video game usage for both children and young adults with autism in terms of treatment approaches with an overall view toward multi-modal therapy. Video games could supplement behavioral therapy by utilizing a digital and online platform that attracts most children with these disorders, thus offering clear advantages in terms of process and results.

Other Mental Health Conditions (Anxiety, Depression, and Post-Traumatic Stress Disorder (PTSD))

For anxiety disorders in children and adolescents, a variety of video games have been developed based on cognitive behavioral therapy (CBT) principles to help young users confront their fears and develop coping strategies. MindLight, for example, is a therapeutic neurofeedback video game specifically designed for children aged eight to 12, aimed at preventing and reducing anxiety symptoms. The game combines immersive gameplay with relaxation techniques and CBT-based challenges. Players navigate a dark, threatening environment while wearing a neurofeedback headband that monitors their level of relaxation through focused breathing and attention control. The game trains children to self-soothe and regulate their anxiety responses by rewarding calm and focused states. Early trials have shown that MindLight is effective in reducing anxiety severity, performing comparably to conventional relaxation training programs [11]. These gamified interventions are particularly valuable for children who may resist or disengage from traditional therapy, as therapeutic techniques are delivered in a covert, engaging manner through the game mechanics.

Fantasy role-playing video games offer a novel approach to treating depression in adolescents. SPARX is a computer game invented by New Zealand researchers as an e-therapy for adolescents suffering from mild to moderate depression. SPARX facilitates a journey through quests and challenges, encouraging gamers to complete CBT modules (such as negative thought identification, problem-solving, and scheduling activities) within a 3D fantasy game world. SPARX was found to be equally as effective as regular face-to-face counselling in alleviating depressive symptoms [12]. After seven weeks of gaming, nearly 44% of adolescents playing SPARX were completely free of depression, which was equivalent to the proportion in those receiving usual care; forms of anxiety and hopelessness also demonstrated improvements, and users reported particular satisfaction with the computer game [12]. These findings suggest that a self-directed therapeutic game may generate clinically significant changes.

Virtual reality (VR) games and simulations are being promoted as exposure therapy appliances for childhood PTSD or PTSD for adults who have not yet recovered from the traumatic effects. VR refers to computer-generated environments experienced through headsets or similar devices, allowing users to interact with immersive, three-dimensional simulations that feel lifelike. The PTSD treatment is frequently used to incrementally manage flashbacks associated with the trauma, little by little, in a managed way to override fear-based reactions. VR exposure therapy used in veterans has exhibited an essential reduction in symptoms found in studies associated with combat-related PTSD: treatments have been effectual for 70% of veterans who were suffering from PTSD after 10 weeks of VR, reaching clinically meaningful reductions in PTSD assessments, compared to 12% of participants receiving conventional care [13]. VR can make exposure more lifelike than imagination, thereby increasing treatment effectiveness through the interactive and immersive nature of this technology. VR can also be adapted to the details of each patient’s trauma story (e.g., details of the virtual environment, the level of stimuli, etc.), thus enabling a truly personalized effect treatment. Apart from the exposure itself, other game-related strategies for PTSD and anxiety include biofeedback games for relaxation training, as well as narrative games that let people reconsider their trauma story from a new angle while playing a game, both of which can help with the emotional processing aspect.

Finding the middle ground

**Figure 2:**
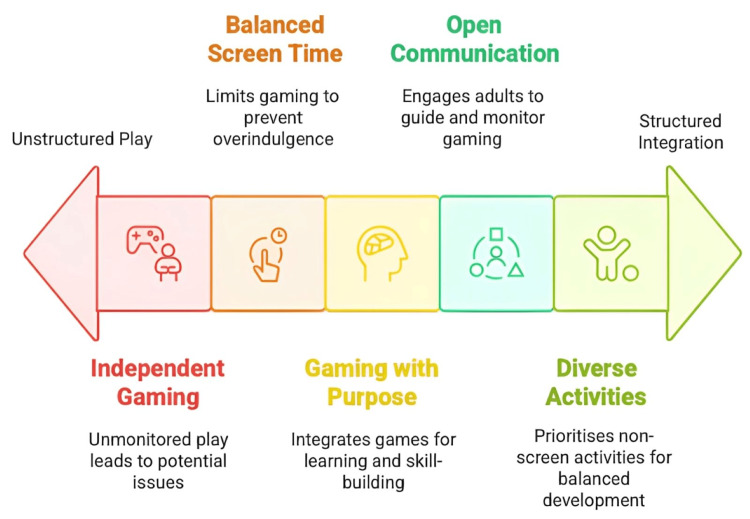
Balancing video game engagement for adolescent well-being and development

Since video games can have both positive and negative influences on the cognitive and emotional well-being of adolescents, it is crucial to find an appropriate balance between their advantages and disadvantages. Banning games is not a viable option, nor is it recommended to leave adolescents to play games independently and without control. As summarized in Figure 2, the following are important recommendations and thoughts for finding the balance:

Establish Appropriate Screen Time

Regularly setting limits on the amount of time spent on gaming is critical for avoiding overindulgence. The American Academy of Pediatrics recommends that children older than two years limit their media exposure to one hour or less per day of high-quality programming, which traditionally refers to educational or age-appropriate television content rather than video games. However, when applied to gaming, the principle may be extended to include games that offer cognitive, social, or therapeutic benefits. Children should also refrain from screens during meals and for one hour before bedtime [14]. Keeping an appropriate daily or weekly gaming limit will guarantee that playtime does not replace homework, chores, or socializing with others. Setting “gaming curfews” - such as no screen time after a certain hour - is also recommended [6].

Curating the Quality

Not all games are created equal. Parents and professionals are encouraged to endorse games that are suitable for the respective age group, have educational value, or help develop cognitive abilities. Many puzzle, strategy, or simulation games have been proven to improve creativity, promote rational thinking, and enhance the amount of knowledge and information obtained through the games. Prosocial games (which have the intention to cooperate or feel empathy for others) increase helpfulness behaviors in real life. Instead, violent games may cause an increase in aggression levels among the users (youth). Looking for specific ratings and explaining what is included in the game helps stick to the learning objectives and value system. Parents may find it challenging to identify which games are developmentally appropriate or therapeutically beneficial, especially if they are not working with a clinician. Several reputable resources can help guide these decisions. For example, Common Sense Media (www.commonsensemedia.org) offers comprehensive, age-based reviews and ratings of games, including educational and social-emotional content. Games for Change (www.gamesforchange.org) highlights socially impactful and learning-oriented games. Parents of children with ADHD or ASD can also consult game-specific recommendations or digital therapeutic tools being evaluated through clinical partnerships or platforms like Akili Interactive or PlayMancer. Accessing these resources can support informed choices about the types of games that best align with children’s cognitive, emotional, and developmental needs.

Gaming With Structure and Purpose

Video games can offer even more benefits when used in structured environments by making the experience an intentional part of the child’s life. In direct learning, teachers utilize educational video games or gamified lessons that are already part of the school curriculum, transforming video gaming into an interactive tool for studying that enhances engagement and retention. In therapy for mental health, clinicians can prescribe certain therapeutic games, monitoring the child’s symptoms over time. Even while playing at home, parents can set the tone that gaming is a privilege that must be earned through homework, chores, or other responsibilities. They can also explain to the child how certain games build skills, such as storytelling to improve new vocabulary in a narrative-heavy game or collaboration in a team-based online multiplayer game. Taking this approach invites self-reflection in children, tempering any compensatory behavior that arises from their misunderstandings or showing why gaming can be seen as an interactive tool related to a sense of immediate pleasure and reinforces the open, positive transfer of skills.

Supervision and Open Communication

It is essential for adults to remain engaged and aware of a child’s gaming experiences. For younger children, co-playing for some time can be an excellent way to establish a connection and help guide their views about the games. Keeping gaming consoles or computers in shared family rooms, rather than bedrooms, makes it easier to ensure supervision. It is essential to look for indicators of problematic gaming, such as an inability to socialize with friends, declining grades in school, or being unable to limit game time independently. If these warnings occur, a non-blaming conversation would be required. In some cases, it may be necessary to invite a specialist to assess the possibility of gaming addiction or other underlying problems (anxiety, ADHD, etc.). It is worth noticing that gaming disorder is believed to affect under 5% of gamers [4], but the faster unhealthy habits are identified, the easier they are to address.

Balance and Activities of Diversity

Suggest developing a balanced schedule where most everyday activities are not connected to screen time. Sports, walking in nature, games played outside, collective hobbies such as music and dance, interactive voluntary service, organizing events, etc., offer a sense of completely different experiences that are in no way similar to computer games. If a child has activities that cannot be substituted by games, the latter will not become the fixation they would otherwise have. This will also help reduce sedentary time and encourage engagement in physical activity. Several families have successfully adopted the concept of a “tech schedule,” such as allowing more video gaming on weekends but maintaining screen-free hours on school days. By doing so, they do not even intend to reduce the demonized role of video games. Instead, they ensure that children develop with a variety of stimuli, with all their functions fulfilled.

Future developments

**Figure 3:**
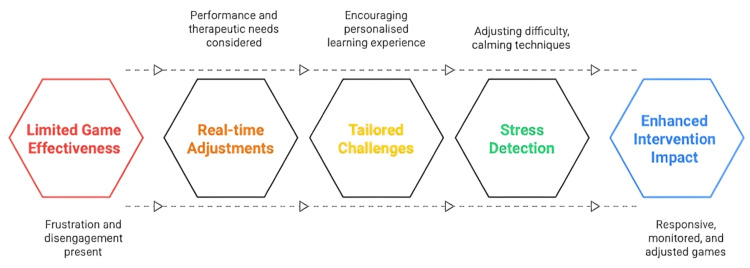
AI-enhanced game for health

Future developments will need to be built on the opportunities for games to contribute to both health and education. Game designers need to incorporate AI into their designs, which can be particularly useful in education and therapy. Serious games that incorporate AI can make real-time adjustments to these factors based on performance and therapeutic needs, ensuring maximum benefit is gained [15]. Learning games can assess students’ understanding of academic subjects and adaptively provide tailored challenges or hints to support learning. An AI therapeutic game may monitor a child’s facial expression or heart rate using sensors to detect signals of stress and react accordingly, adjusting game difficulty or implementing calming techniques at the appropriate times. Game-based interventions can be more effective when they incorporate responsive elements that prevent frustration and disengagement, as illustrated in Figure 3. This would enable health professionals to monitor and adjust as needed, using the exact mechanisms employed for medications or tutoring sessions.

## Conclusions

This narrative review set out to examine the dual role of video games in children and adolescents, focusing on their cognitive effects and emerging use as digital therapeutic tools. The evidence indicates that specific genres of video games - such as action, puzzle, and strategy games - can enhance cognitive functions, including attention, working memory, problem-solving, and visual-spatial skills. Therapeutic games, including EndeavorRx, SPARX, and MindLight, demonstrate promising outcomes in managing symptoms of ADHD, anxiety, and depression in youth. However, excessive or unstructured gaming is associated with risks, including attention difficulties, sleep disruption, reduced academic performance, and displacement of physical or social activities. These findings highlight the importance of appropriate screen-time limits, game selection, and active parental or clinical guidance.

Clinicians should consider including gaming habits in routine pediatric assessments and offer balanced, evidence-based guidance to families. Future longitudinal research is needed to investigate the long-term effects of gaming and its integration into educational and therapeutic contexts. With thoughtful implementation, video games can serve as meaningful tools to support learning and mental health in young populations.
